# EVALUATION OF A REMINISCENCE AND PHOTO-IMAGERY WALKING PROGRAM FOR BLACK OLDER ADULTS

**DOI:** 10.1093/geroni/igae098.1268

**Published:** 2024-12-31

**Authors:** Boeun Kim, Lia Kaluna, Meshack Otewa, Basia Belza, Raina Croff

**Affiliations:** University of Iowa, Iowa, United States; University of Wisconsin Health University Hospital, Madison, Wisconsin, United States; University of Washington, Seattle, Washington, United States; University of Washington, Seattle, Washington, United States; Oregon Alzheimer’s Disease Research Center, Oregon Health & Science University, Portland, Oregon, United States

## Abstract

The purpose of this study was to pilot the Sharing History through Active Reminiscence and Photo-Imagery (SHARP) program which honors Black history and health through culturally celebratory walking routes in the Puget Sound area. Participants were recruited from a senior center. Thirty-one healthy and mildly cognitively impaired Black Americans completed triadic walking three times a week for 4 weeks. Participants used the SHARP walking application on a group tablet device, and back-up paper routes, to navigate twelve themed, 1-mile looped routes with GPS-triggered local historical images of Black life and culture to prompt conversational reminiscence during walks. Route themes were Childhood Health, Black Advocacy, Community Ties, Building Community, Arts, Entertainment, and Education. Focus groups at the end of the study captured participant experiences, challenges, and preferences. Pre/post measures captured blood pressure, self-rated health, and depressive symptoms. Paired t-tests were performed on pre/post outcomes, and thematic coding was applied to focus group data. Mean age was 71.8 years and the majority (93%) were female. Mean self-rated health improved and depression symptoms decreased, but not statistically significant. Blood pressure had little to no change. End-study focus groups revealed that participants were motivated by and benefited from social engagement with their walking groups. Walks simultaneously motivated people to exercise and facilitated reminisce about lived experiences in the neighborhood, including gentrification. Participants identified confusing prompts, local construction, terrain, and technology as barriers to the current application of the program. Participants made suggestions for future route themes and to improve program accessibility and clarity.

